# Assessment of Nutritional Value and Maillard Reaction in Different Gluten-Free Pasta

**DOI:** 10.3390/foods12061221

**Published:** 2023-03-13

**Authors:** Maria Cristina Messia, Francesca Cuomo, Michela Quiquero, Vito Verardo, Emanuele Marconi

**Affiliations:** 1 Department of Agricultural Environmental and Food Science, University of Molise, Via F. de Sanctis, 86100 Campobasso, Italy; 2 Department of Nutrition and Food Science, University of Granada, Campus of Cartuja s/n, 18071 Granada, Spain; 3 Institute of Nutrition and Food Technology ‘José Mataix’, Biomedical Research Centre, University of Granada, Avd. Conocimiento s/n, 18100 Armilla, Spain; 4 Research Unit of Food Science and Human Nutrition, Department of Science and Technology for Humans and the Environment, Università Campus Bio-Medico di Roma, Via Álvaro del Portillo 21, 00128 Rome, Italy

**Keywords:** gluten-free pasta, furosine, protein chemical score, pseudocereals, pulse, maltulose, Maillard reaction

## Abstract

Evaluating the nutritional quality and thermal damage effects of gluten-free foods is essential to ensure that people with gluten intolerance or celiac disease can safely meet their needs. In this work, fifteen different commercial gluten-free pasta samples made from cereals, pseudocereals, and pulses, alone or in mixed combinations, were analyzed to assess their nutritional value, essential amino acids composition, and protein chemical score. The occurrence of the Maillard reaction was investigated, and the levels of heat treatment markers (furosine, maltulose, hydroxymethylfurfural, and glucosylisomaltol) were determined. Analysis of the furosine values showed that pasta made with the same raw materials can have different degrees of thermal damage. There was no evidence of the Maillard reaction progressing in the advanced phase in any of the samples tested. Finally, the correlation between maltulose and furosine levels demonstrated the usefulness of combining the two markers to assess the extent of thermal damage.

## 1. Introduction

A gluten-free (GF) diet is the most important requirement for people affected by celiac disease [1] and gluten intolerance [2]. In recent years, the market of gluten-free products has been boosted by the increasing demand for these products, which meet not only the medical requirements but also the health perception of millions of customers [3]. Although they are also in demand by consumers without celiac disease, gluten-free products are, in any case, destined for a limited market share, given their market price (which is more than double that of products containing gluten) [4]. 

Pasta represents an important and popular product, also in a GF diet, due to its long shelf-life and ease of use. For these reasons, many different types of GF pasta, made from different raw materials such as rice, pseudocereals, corn, legumes etc.) can be found on the market today [5]. The main difficulties in the production of GF pasta lie in the development of a structure with the functional properties that gluten ensures on cooked pasta [6]. Excellent sensory, nutritional and cooking attributes are, indeed, important to avoid excluding people with celiac disease and gluten intolerance from a nutritive and psychological point of view. 

An important aspect of diets, in general, is the quality of nutrients. In the protein fraction, the amino acid composition is important to assess the quality of protein intake [7]. For example, legume proteins are characterized by low content of sulfur-containing amino acids, while cereals are low in lysine. It is, therefore, desirable to formulate GF pasta without amino acid deficiencies. 

Pasta drying is a fundamental step in pasta production, applied to bring the product to a moisture content of less than 12.5% as required by the Italian law [8]. This type of process extends the shelf life of pasta by reducing water activity and inactivating enzymes. In addition, heat can cause “thermal damage” generally associated with the Maillard reaction (MR), also known as a non-enzymatic browning [9,10]. Among the compounds that can be used as markers of the drying process, the descriptors of the early and advanced phases of MR are of particular importance [10,11,12,13,14,15]. MR involves numerous cascade reactions during food production and transformation processes. The course of the reaction depends on the initial composition of the food matrix, the extent of heat treatment applied, water activity, pH and moisture [16]. MR takes place when reducing sugars and the amino groups of free amino acids and proteins interact. Among the amino acids, lysine is the most susceptible to MR and is irreversibly blocked in the newly formed Amadori compound (AC) in the initial phase of MR, which subsequently leads to nutritional damage to foods. AC can be indirectly evaluated through the formation of the ε-N-2-furoylmethyl-L-lysine (furosine, FUR), an unnatural amino acid formed with the acid hydrolysis of AC [17,18]. Thus, FUR is an important indicator of the early stage of MR. Together with FUR, other indicators of the MR should also be considered, such as hydroxymethylfurfural (HMF), which is formed in the second step of the reaction after the decomposition of AC into dicarbonyl compounds, and glucosyl isomaltol (AGPF), which is formed by the degradation of AC and whose concentration correlates with that of maltose [10,19]. In addition, maltulose can be formed by the isomerisation of maltose during the heat treatment [20].

The literature on GF pasta mainly focuses on the study of the structural properties of the raw/cooked commercial [21,22] and experimental pasta [23,24] and on the cooking quality. Other recent studies, on the other hand, have mainly focused on pasta formulation improvement with highly nutritious raw materials [6,25,26,27,28,29]. There are very few studies dealing with the heat damage caused by the drying process of GF pasta, such as that of Gasparre et al. [30] who investigated commercial GF-dried spaghetti. 

With this background, in the present study, besides investigating the nutritional value of commercial GF short pasta by composition, amino acid analyses and chemical score calculation, the effect of the heat treatment was evaluated by checking the progress of MR by assessing the markers of the early (FUR) and advanced phase (HMF and AGPF) of the reaction. 

## 2. Materials and Methods

### 2.1. Materials

Fifteen types of GF pasta samples, short shape, were purchased from local supermarkets. The composition of pasta, as indicated by the manufacturer on labels, and the coding of samples are given in Table 1. 

### 2.2. Determination of Moisture, Protein, Fat and Fibre of Gluten-Free Pasta

Prior to physicochemical analysis, pasta was ground through a refrigerated mill (Ika A10, Ika Fisher Scientific, Staufen, Germany). Moisture and fat content were determined according to ICC methods 109/1, 136 [31]. Protein content was determined using a Leco nitrogen determiner, model FP 528 (Leco Corp., St. Joseph, MI, USA) using the Dumas combustion method, AACC method 46-30.01 (N × 6.25) [32]. Dietary fibre was determined according to the AACC Method 32-05.01 [32]. Total starch was determined according to AACC Method 76-13.01 [32] using a dedicated test kit (K-TSTA, Neogen Megazyme, Lansing, MI, USA). 

### 2.3. Analysis of Amino Acid 

Essential amino acids (EAA) were evaluated on commercial GF pasta. Samples were treated by acid and alkaline hydrolysis, as previously reported [33]. Briefly, for acid hydrolysis, an amount of ground pasta corresponding to a protein content of 25 mg was hydrolyzed with 25 mL of 6 N HCl at 110 °C for 24 h. The samples were then filtered, evaporated to dryness and dissolved in 0.1 N HCl. In the alkaline hydrolysis, for the determination of tryptophan, a sample containing 10 mg of protein was mixed with 1 mL of distilled water, 5 mL of 10 N NaOH and 4 mL of distilled water and then hydrolyzed for 18 h at 110 °C. After cooling, the sample was neutralized by adding 6 N HCl, evaporated to dryness, and dissolved in 0.1 N HCl. All samples were diluted 50 or 100-fold with ultra-pure water and analyzed by an ICS6000 chromatographic system (Thermo Fisher Scientific S.p.A, Milano, Italy). Separation was performed with an Aminopac PA10 analytical column (250 × 2 mm, 8.5 µm particle size) (Thermo Fisher Scientific S.p.A, Milano, Italy). The conditions of the chromatographic separation of the amino acids are illustrated in Table S1.

The chemical score value (CS) was calculated according to the Food and Agriculture Organization using the recommended amino acid scoring pattern for older children, adolescents and adults [7]. The CS is given by the ratio between the amount of a given EAA in 1 g of protein in the food matrix to the amount of the same amino acid in 1 g of the reference protein ×100.

### 2.4. Determination of Sugars

The samples were prepared according to the procedure described by Berrios [34]. An HPAEC-PAD Dionex ICS-6000 system (Thermo Fisher Scientific Spa, Milan, Italy) was used for the analysis of sugars. Separation was performed using a CarboPac PA100 analytical column (2 × 250 mm) equipped with a pre-column at a temperature of 25 °C and a flow rate of 0.25 mL/min. Detection was performed with an electrochemical detector. The eluents used were A = water, B = 100 mM NaOH, and C = 100 mM NaOH + 120 mM Sodium Acetate. The gradient flow adopted was as follows: 15% B and 85% A for 35 min; 18% B, 12% C and 70% A for up to 50 min; 25% B, 25% C and 50% A, up to 50.1 min; 15% B and 85% A up to 60 min.

### 2.5. Determination of Furosine (FUR) 

A quantity of finely ground pasta, corresponding to about 30–70 mg of protein, was hydrolyzed with 8 mL HCl 8 N at 110 °C for 23 h in Pyrex tubes under nitrogen. Samples were analyzed according to the procedure developed by Resmini et al. [18] using an HPLC system (UltiMate 3000, Thermo Fisher Scientific, S.p.A, Milano, Italy) coupled to a variable wavelength detector at 280 nm. Analytical separation was made using a furosine-dedicated column (Grace, Reading, Berkshire, UK). The furosine standard was purchased from the Neosystem Laboratoire (Strasbourg, France).

### 2.6. Determination of Hydroxymethylfurfural (HMF) and Glucosyl Isomaltol (AGPF)

Firstly, 250 mg of finely ground pasta was placed in 1 mL sodium borate buffer (0.1 M, pH 8.2) at 30 °C for 30 min. This was followed by centrifugation at 5500 g for 20 min at 5 °C. Then, 0.6 mL of supernatant was mixed with 30 µL of 15% *w/v* potassium ferrocyanide solution (Carrez I) and 30 µL of 30% *w/v* zinc sulphate heptahydrate solution (Carrez II). The mixed samples were stored at 4 °C for 1 h and then centrifuged at 5500 g for 20 min at 5°C. The supernatant was analysed by HPLC (UltiMate 3000, Thermo Fisher Scientific) using a column NOVA-PAK C18 60 Å (300 mm × 3.9 mm × 4 µm) using isocratic elution. The mobile phase consisted of water:acetonitrile (97:3), with a flow rate of 0.7 mL/min. HMF and AGPF were detected at 280 nm. Quantification was performed using a standard calibration curve of HMF (LOQ 0.03 mg/kg; LOD 0.002 mg/kg) and AGPF (LOQ 0.39 mg/kg; LOD 0.19 mg/kg), respectively. Linearity range of (1) HMF 0.03 mg/L–0.50 mg/L; (2) AGPF 0.2 mg/kg–12.5 mg/kg.

### 2.7. Statistical Analysis

Each sample analysis was made in triplicate. Results are reported as mean ± standard deviation. Analysis of variance (ANOVA) and Tukey tests were performed with the SPSS Version 23.0 (IBM SPSS Statistics, Armonk, NY, USA), and differences at *p* < 0.05 were considered significant.

## 3. Results and Discussion

### 3.1. Evaluation of Pasta Composition

Of the pasta samples analyzed, 40% were made exclusively of legume flours (P1 to P6) (peas, red lentils, lentils, chickpeas), only two samples were prepared with 100% corn (P11) or 100% buckwheat (P7), while the rest contained a mixture of cereals (corn, rice, and teff), pseudocereals (amaranth, buckwheat, and quinoa) and legume flours. Only sample P10 showed the presence of emulsifiers (mono- and diglycerides of fatty acids) in the ingredients list on the label. Although some of the samples contained the same ingredients, a significant difference in their nutritional composition was found (Table 2).

The use of legume flour helped to increase the protein and fibre content of pasta. The fibre content can also be increased by using ingredients such as amaranth, quinoa and teff, as in P12, which are usually wholegrain milled or by adding bamboo fibre, as in P13. 

The use of legume flour or a pseudocereal (quinoa, buckwheat, amaranth) as a basic ingredient shows the company’s intention to produce pasta with a higher protein content and an improved biological value of the proteins. It is known that the lysine deficiencies of cereals can be compensated by adding flours from legumes or pseudocereals (vide infra), also in pasta formulations for people suffering from celiac disease or gluten intolerance. The samples studied had variable protein content with an average concentration of 15.6% ranging from 5.06 to 29.53%, indicating a high variability. The differences found were attributed to the variability in the composition of the ingredients used. 

### 3.2. Amino Acid Analysis and Chemical Score

The biological value of proteins is closely related to their EAA composition, and the quali-quantitative amino acid profile of commercial pasta can be influenced by the different raw materials and formulations.

In GF pasta, whose essential amino acid composition is listed in Table 3, pasta made only from legumes (P1 to P6) had an EAA profile very similar to that of legumes [35,36], with methionine and cysteine being the limiting amino acids. Pasta P7 made from buckwheat had no limiting amino acid, unlike the other pasta, from P8 to P14, which had lysine as the limiting amino acid. 

Pasta P11 was made from corn flour only, and the EAA composition was similar to corn flour, as expected [37], with a CS of 55. Pasta P13, made from more than 90% of cereal flour (corn and rice), was also low in lysine, with a CS of 57. A slight increase of the CS to a value of 64, with lysine still as the limiting amino acid, was observed in P10, where the presence of brown rice partially improved the lysine content. The formulation of P8 contained a mixture of cereals and pseudocereals. The latter added at only 15%, caused an improvement of the CS to a value of 70. Further improvement of the CS was observed in P9 and P12; 10% amaranth and 5% quinoa flours increased the CS in P12 to 71, while 25% quinoa flour in P9 helped to achieve a CS value of 83. 

In P14 and P15 pasta, cereal flours were combined with legume flours, which increased the CS value of pasta. The CS was 82 in P14 with 30% pea flour and 88 in P15 with lentil flour.

### 3.3. Sugar Composition Assessment

Carbohydrates are the principal component in most types of pasta, from those made from semolina to special pasta (see also Table 2), and therefore their analysis is interesting from a nutritional point of view. Reducing sugars, such as monosaccharides like galactose, glucose and fructose, make up only a small part of carbohydrates but still are of interest because they contribute to the formation of AC, together with the amino acid in MR. 

This experimental work identified some of the sugars that are common to the different types of pasta: monosaccharides (galactose, glucose and fructose) and disaccharides (sucrose, maltulose and maltose). Oligosaccharides, such as raffinose and stachyose, were also found in pasta prepared with legume flours [34] but have not been quantified in this context. Furthermore, disaccharides, such as sucrose, maltulose and oligosaccharides, such as raffinose and stachyose, do not participate in MR, as they are not reducing sugars.

Among the quantified sugars, as shown in Table 4, sucrose is present in higher concentrations (average value = 1738 mg/100 g), although interesting amounts of reducing sugars were found, such as galactose (average value = 142 mg/100 g), glucose (average value = 184 mg/100 g) and fructose (average value = 145 mg/100 g) which may be directly involved in MR. Maltose, with an average value of 103 mg/100 g, and maltulose (Table 5) were also found in all samples. The varying amount of maltose can be attributed to the different types of raw materials used for the pasta formulations. 

### 3.4. Assessment of Heat Treatment Incidence 

In the production of conventional pasta, the combination of short times/high temperatures allows the production of pasta with excellent cooking properties, even when using low-quality semolina, but with the risk of brown pigment formation as products of MR [38]. FUR, HMF, AGPF and maltulose are recognized markers to assess the thermal damage caused by heat treatment and, thus, by the occurrence of the MR [10,11].

The occurrence of MR may depend on several factors, apart from the raw materials and the temperature applied during the drying process, and in the case of GF pasta, the use of different technologies should also be considered. Indeed, compared to traditional pasta production, the process can be modified according to the different raw materials used in order to promote the formation of a suitable structure that will give a good product after cooking [5]. 

The typical process used for low-protein raw materials is hydrothermal treatment. Here, the reorganization of the retrograded starch provides a suitable network during cooking. Pre-gelatinization of starch is an alternative when other ingredients are also included in the pasta formulation. Extrusion cooking is instead a process in which heat is applied under pressure. In all these cases, the raw materials or semi-finished products are also treated with heat before drying. 

The FUR value of semolina pasta has been demonstrated to be influenced by drying cycles. FUR levels can vary from 45 to 200 mg/100 g protein for pasta made with a combination of low temperature and long time, to values from 400 to 600 mg/100 g protein and more, for combinations of high temperature and short time [15]. In general, short pasta made from 100% semolina is characterized by lower FUR values (<200 mg/100 g protein) compared to long pasta due to the different effects of drying cycles on the different shapes [20]. Very little information is available in the literature on the evaluation of FUR in the GF pasta [30]. As reported in Table 5, the average value in the current experiment was about 192 mg/100 g protein, with a range varying between 18.9 and 787.6, proving the wide variability in both drying conditions and raw materials.

Specifically, the highest FUR values were found in samples P2 and P3 (602 and 788 mg/100 g protein, respectively) made from 100% red lentil flour and 100% chickpea flour. These two GF pasta reached FUR values rarely found in short semolina pasta, even when high temperatures are applied [20]. On the other hand, the lowest values were found in samples P1 and P7 (21 and 19 mg/100 g protein, respectively), which were made with 100% green pea flour and 100% buckwheat flour. Apart from the values found in samples P2 and P3, the data obtained are generally in agreement with those previously reported by Gasparre et al. [30] (range 19–134 mg/100 g protein) and lower than the values found in conventional semolina pasta (up to 450 mg/100 g protein) [39]. For the same ingredient (see samples P1, P4 and P6), the different values of FUR can be attributed to different factors: (a) use of native/heat-treated/pre-gelatinized flours; (b) hydrothermal treatment; (c) extrusion-cooking treatment; (d) time/temperature combination during drying [5]. 

From the obtained data, it can be deduced that even in GF pasta, high FUR levels can be reached, which are even higher than in pasta made from 100% semolina (see FUR levels of samples P2 and P3). The high value of FUR in legumes can be attributed to the simultaneous presence of proteins (i.e., lysine) and reducing sugars in such molar ratios that they favour the initiation of MR and lead to the formation of AC. The development of MR has important consequences associated with the reduction of the nutritional value of pasta due to the involvement of amino acids and, in particular, the EAA lysine. 

Maltulose, whose levels are shown in Table 5, is an isomer of maltose. It is the only sugar produced by the drying process and was previously used, in combination with FUR, to evaluate the thermal damage of conventional dried pasta [20]. However, there are no studies in the literature on the relationship between FUR and maltulose in GF pasta. 

**Figure 1:**
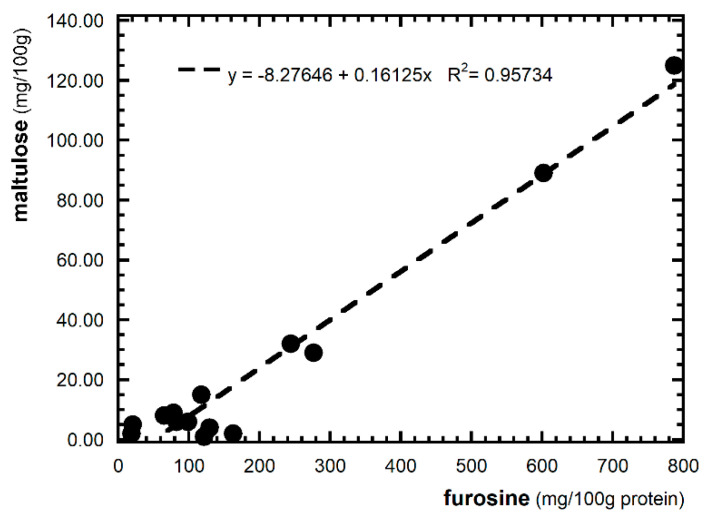
Correlation between FUR and maltulose.

The levels of maltulose in GF pasta were found to be in amounts averaging 23 mg/100 g. Interestingly, the highest values of maltulose were found in pasta that also had the highest value of FUR (P2, P3, P5, P12). The correlation between FUR and the maltulose values found in the analyzed GF pasta is shown in Figure 1. The correlation confirms that maltulose, together with FUR, can be used as a process marker for dried pasta to verify the thermal damage [20].

HMF is an intermediate product of MR, which is used to evaluate the extent of thermal treatment but also derives from the dehydration of hexoses [40]. In the GF pasta samples investigated, HFM was detected in very low concentrations and only in a few of the samples. However, considering the average levels of FUR in the different samples, the observed low HMF levels were the expected ones. In P2 and P3, which had high levels of FUR, the low amount of HMF indicated that MR did not progress strongly to the advanced phases.

Along with FUR and HMF, the content of AGPF was also examined. The result was that AGPF was not detectable in any of the samples (<LOD = 0.19 mg/kg). This result was in agreement with both the values of FUR and maltose concentration (as shown in Table 5) since, as already stated, the formation of AGPF is strongly correlated with the presence of maltose. The detected maltose levels were indeed much lower than those commonly found in traditional semolina pasta (6 to 553 mg/100 vs 1300 to 2500 mg/100 g) [11]. 

As noted in previous studies [11,17], the lysine loss characterizing semolina pasta with maximum FUR levels of 100 mg/100 g protein is between 3 and 7%; for FUR levels < 400 mg/100 g protein, a maximum loss of lysine up to 30% occurs, and for FUR up to about 700 mg/100 g protein blocked lysine is between 30% and 50%. Considering the average FUR levels found in the GF pasta and the EAA profile, it can be deduced that the GF pasta studied here, apart from P2 and P3 samples, were subject to reduced nutritional damage. 

## 4. Conclusions

Nowadays, a gluten-free diet is easier to achieve thanks to the wide range of products available on the market. Assessing the quality of gluten-free foods is important: (1) to ensure that celiac people can fully satisfy their needs and (2) to evaluate the possible changes that the products may undergo during the manufacturing processes.

The results of this study show that the Maillard reaction can also occur in gluten-free pasta. The amino acids and reducing sugars in the flours from legume and pseudo-cereal used for the production of commercial gluten-free pasta were present in such molar ratios that they favored the triggering of the Maillard reaction. 

The study showed that the levels of furosine and maltulose, used as markers of the intensity of the heat treatment, can reach high values (which are also higher than those in pasta made from 100% semolina). The good correlation between the maltulose and furosine values supports the possibility of using the two indices alone or in combination to assess the intensity of heat treatment, as has been performed for conventional pasta. The advanced phase markers of the Maillard reaction (hydroxymethylfurfural and glucosyl isomaltol) were present in negligible or undetectable amounts. 

Moreover, the combination of different, properly selected raw materials helps to formulate products with high protein chemical scores. 

## Figures and Tables

**Table 1:** List of gluten-free pasta samples.

Sample	Ingredients
*Gluten-Free Pasta—100% legume flour*	
P1	100% Green Pea Flour
P2	100% Red Lentil Flour
P3	100% Chickpea Flour
P4	100% Green Pea Flour
P5	100% Lentil Flour
P6	100% Green Pea Flour
*Gluten-Free Pasta—Cereal, pseudocereal and legume flours (100% or mixed)*	
P7	100% Buckwheat Flour
P8	75% Corn Flour, 10% Rice Flour, 10% Buckwheat Flour, 5% Quinoa Flour
P9	75% Rice Flour, 25% Quinoa Flour
P10	36% Brown Rice Flour, 32.5% Yellow Corn Flour, 20% White Corn Flour, 8% Rice Flour, 3% Potato Starch, Emulsifiers (mono and diglycerides of fatty acids)
P11	100% Corn Flour
P12	Corn Flour, Rice Flour, 10% Amaranth Flour, 5% Teff Flour, 5% Quinoa Flour
P13	Corn Flour, Rice Flour, 8% Bamboo Fibers
P14	Corn Flour, 30% Pea Flour, Rice Flour
P15	Corn Flour, 30% Red Lentil Flour, Rice Flour

**Table 2:** Average nutritional values (g/100 g dw) of gluten-free pasta samples. Different letters in columns indicate statistically significant differences (*p* < 0.05).

Sample	Moisture (%)	Fat	Fibre	Protein	Total Starch
*Gluten-Free Pasta—100% legume flour*					
P1	10.8 ± 0.3 ^abc^	2.02 ± 0.03 ^de^	9.91 ± 0.16 ^g^	24.37 ± 0.03 ^l^	58.2 ± 1.23 ^c^
P2	10.6 ± 0.2 ^ab^	1.92 ± 0.15 ^de^	8.49 ± 0.15 ^f^	26.60 ± 0.13 ^n^	54.2 ± 0.98 ^bc^
P3	11.0 ± 0.1 ^abc^	6.98 ± 0.12 ^i^	15.78 ± 0.12 ^l^	24.17 ± 0.06 ^il^	46.9 ± 1.03 ^a^
P4	10.5 ± 0.3 ^a^	2.63 ± 0.05 ^g^	8.70 ± 0.15 ^f^	25.18 ± 0.06 ^m^	58.9 ± 0.87 ^c^
P5	10.7 ± 0.2 ^abc^	1.62 ± 0.11 ^c^	7.18 ± 0.10 ^e^	29.53 ± 0.22 ^o^	56.2 ± 0.25 ^bc^
P6	11.1 ± 0.1 ^bc^	6.78 ± 0.12 ^i^	12.54 ± 0.15 ^h^	23.97 ± 0.03 ^h^	52.7 ± 0.77 ^b^
*Gluten-Free Pasta—Cereal, pseudocereal and legume flours (100% or mixed)*					
P7	10.6 ± 0.2 ^ab^	3.24 ± 0.07 ^h^	5.06 ± 0.10 ^d^	9.81 ± 0.02 ^f^	73.4 ± 2.10 ^e^
P8	10.8 ± 0.1 ^abc^	1.82 ± 0.20 ^cd^	2.83 ± 0.13 ^b^	9.30 ± 0.08 ^e^	82.6 ± 2.35 ^fg^
P9	11.2 ± 0.1 ^c^	1.21 ± 0.03 ^b^	1.11 ±0.02 ^a^	9.40 ± 0.01 ^e^	84.0 ± 2.11 ^fg^
P10	10.9 ± 0.1 ^abc^	2.22 ± 0.10 ^f^	3.44 ± 0.12 ^c^	7.69 ± 0.04 ^c^	81.0 ± 1.98 ^f^
P11	10.6 ± 0.2 ^ab^	0.91 ± 0.03 ^a^	1.01 ± 0.01 ^a^	5.06 ± 0.04 ^a^	86.7 ± 2.00 ^g^
P12	10.5 ± 0.3 ^a^	3.24 ± 0.02 ^h^	13.45 ± 0.12 ^i^	8.80 ± 0.06 ^d^	64.4 ± 1.54 ^d^
P13	10.9 ± 0.2 ^abc^	1.82 ± 0.02 ^cd^	9.91 ± 0.31 ^g^	7.38 ± 0.01 ^b^	72.3 ± 2.14 ^e^
P14	10.7 ± 0.1 ^abc^	2.02 ± 0.01 ^de^	3.74 ± 0.05 ^c^	10.92 ± 0.01 ^g^	73.9 ± 2.22 ^e^
P15	11.1 ± 0.1 ^bc^	2.12 ± 0.04 ^ef^	3.34 ± 0.05 ^c^	12.03 ± 0.01 ^i^	73.4 ± 2.36 ^e^
Mean	10.8	2.70	7.10	15.6	67.9
Min-max	10.5–11.2	0.91–6.98	1.11–15.78	5.06–29.53	46.9–86.7

**Table 3:** EAA (mg/g protein) CS and limiting amino acid of GF pasta. Different letters in rows indicate statistically significant differences (*p* < 0.05).

**EAA ^1^**	*Gluten-Free Pasta—100% Legume Flour*						*Gluten-Free Pasta—Cereal, Pseudocereal and Legume Flours (100% or Mixed)*									**FAO Amino Acid Scoring Pattern (mg/g Protein)** [7]
	**P1**	**P2**	**P3**	**P4**	**P5**	**P6**	**P7**	**P8**	**P9**	**P10**	**P11**	**P12**	**P13**	**P14**	**P15**	
**His**	2.59 ± 0.01 ^(a)^	2.64 ± 0.02 ^(a)^	2.46 ± 0.02 ^(a)^	2.37 ± 0.12 ^(a)^	2.35 ± 0.32 ^(a)^	2.54 ±0.3 ^(a)^	5.18 ± 0.53 ^(b)^	2.94 ±0.07 ^(a)^	2.50 ± 0.06 ^(a)^	2.56 ± 0.19 ^(a)^	2.66 ± 0.12 ^(a)^	2.82 ± 0.08 ^(a)^	2.54 ±0.17 ^(a)^	2.64 ±0.23 ^(a)^	2.70 ± 0.06 ^(a)^	1.6
**Ile**	3.69 ± 0.02 ^(de)^	2.94 ± 0.06 ^(ab)^	3.48 ± 0.09 ^(bcd)^	3.39 ± 0.21 ^(bcd)^	2.61 ± 0.12 ^(a)^	3.60 ± 0.22 ^(cde)^	3.33 ± 0.12 ^(bcd)^	3.27 ±0.23 ^(bcd)^	4.11 ± 0.22 ^(e)^	3.45 ± 0.41 ^(bcd)^	3.00 ± 0.23 ^(ab)^	3.06 ± 0.16 ^(abc)^	3.15 ± 0.12 ^(abcd)^	3.36 ± 0.08 ^(bcd)^	3.18 ±0.23 ^(abcd)^	3
**Leu**	7.14 ± 0.03 ^(bc)^	5.73 ± 0.21 ^(ab)^	6.77 ± 0.22 ^(b)^	6.59 ± 0.30 ^(ab)^	5.12 ± 0.70 ^(a)^	7.02 ± 0.72 ^(bc)^	6.04 ± 0.56 ^(ab)^	10.80 ± 0.67 ^(ef)^	8.48 ±0.44 ^(cd)^	10.00 ±0.57 ^(def)^	11.47 ±0.81 ^(f)^	9.70 ±0.77 ^(de)^	10.74 ±0.68 ^(ef)^	10.07 ±0.22 ^(def)^	9.76 ± 0.45 ^(de)^	6.1
**Lys**	6.77 ± 0.10 ^(ef)^	5.28 ± 0.10 ^(d)^	4.61 ± 0.12 ^(cd)^	6.19 ± 0.43 ^(e)^	6.48 ± 0.12 ^(ef)^	6.62 ±0.44 ^(ef)^	7.01 ± 0.53 ^(f)^	3.36 ± 0.23 ^(ab)^	3.98 ± 0.13 ^(bc)^	3.07 ± 0.17 ^(a)^	2.69 ±0.11 ^(a)^	3.41 ±0.09 ^(ab)^	2.74 ± 0.16 ^(a)^	3.98 ±0.08 ^(bc)^	4.22 ±0.32 ^(c)^	4.8
**Met**	0.87 ± 0.01 ^(a)^	0.74 ± 0.02 ^(a)^	0.83 ± 0.04 ^(a)^	0.80 ± 0.01 ^(a)^	0.66 ± 0.03 ^(a)^	0.86 ± 0.11 ^(a)^	1.56 ± 0.08 ^(b)^	2.06 ± 0.15 ^(d)^	2.52 ±0.22 ^(e)^	2.09 ±0.11 ^(d)^	1.97 ± 0.04 ^(cd)^	2.20 ± 0.11 ^(d)^	2.05 ± 0.10 ^(d)^	1.75 ± 0.07 ^(bc)^	1.72 ± 0.09 ^(bc)^	SAA * 2.3
**Cys**	1.13 ± 0.02 ^(b)^	0.77 ± 0.01 ^(ab)^	1.07 ± 0.07 ^(b)^	1.04 ± 0.12 ^(ab)^	0.69 ± 0.01 ^(a)^	1.11 ± 0.22 ^(b)^	2.66 ± 0.12 ^(f)^	1.93 ± 0.15 ^(de)^	1.63 ± 0.09 ^(cd)^	1.60 ± 0.17 ^(cd)^	1.84 ± 0.08 ^(cde)^	2.05 ± 0.25 ^(e)^	1.79 ±0.08 ^(cde)^	1.65 ±0.05 ^(cd)^	1.56 ± 0.08 ^(c)^	
**Phe**	4.46 ± 0.03 ^(cd)^	3.71 ± 0.10 ^(ab)^	4.22 ± 0.13 ^(bcd)^	4.08 ± 0.21 ^(bc)^	3.30 ± 0.23 ^(a)^	4.36 ± 0.32 ^(bcd)^	4.66 ±0.33 ^(cd)^	4.85 ± 0.07 ^(d)^	5.74 ± 0.12 ^(e)^	4.84 ±0.41 ^(d)^	4.58 ±0.27 ^(cd)^	4.49 ±0.14 ^(cd)^	4.78 ± 0.09 ^(d)^	4.77 ±0.23 ^(d)^	4.62 ± 0.23 ^(cd)^	AAA ** 4.1
**Tyr**	2.70 ± 0.05 ^(bcdef)^	2.30 ± 0.07 ^(ab)^	2.56 ± 0.09 ^(abcd)^	2.47 ± 0.13 ^(abc)^	2.05 ± 0.15 ^(a)^	2.64 ± 0.11 ^(bcde)^	4.86 ±0.33 ^(h)^	3.49 ±0.28 ^(g)^	3.00 ±0.18 ^(cdefg)^	3.36 ±0.09 ^(g)^	3.28 ±0.31 ^(g)^	3.22 ±0.12 ^(fg)^	3.15 ± 0.18 ^(efg)^	3.12 ±0.22 ^(efg)^	3.04 ± 0.13 ^(defg)^	
**Thr**	3.40 ± 0.01 ^(cd)^	2.80 ± 0.09 ^(ab)^	3.23 ± 0.10 ^(bc)^	3.10 ± 0.22 ^(bc)^	2.50 ± 0.12 ^(a)^	3.33 ± 0.28 ^(bcd)^	3.88 ± 0.41 ^(d)^	3.55 ±0.22 ^(cd)^	3.45 ±0.22 ^(cd)^	3.43 ±0.17 ^(cd)^	3.40 ±0.12 ^(cd)^	3.20 ± 0.03 ^(bc)^	3.30 ±0.11 ^(bc)^	3.43 ± 0.25 ^(cd)^	3.30 ±0.02 ^(bc)^	2.5
**Val**	4.16 ± 0.03 ^(abcd)^	4.32 ± 0.08 ^(abcde)^	3.96 ± 0.30 ^(ab)^	3.80 ± 0.14 ^(a)^	3.84 ± 0.22 ^(ab)^	4.08 ± 0.31 ^(abc)^	4.40 ± 0.21 ^(abcde)^	4.84 ± 0.04 ^(ef)^	5.88 ± 0.07 ^(g)^	5.08 ±0.11 ^(f)^	4.60 ±0.36 ^(cdef)^	4.44 ±0.09 ^(bcde)^	4.80 ± 0.33 ^(ef)^	4.72 ± 0.12 ^(def)^	4.84 ± 0.12 ^(ef)^	4
**Trp**	0.91 ± 0.01 ^(e)^	0.80 ± 0.02 ^(bcde)^	0.86 ± 0.03 ^(de)^	0.84 ± 0.01 ^(cde)^	0.71 ± 0.02 ^(abc)^	0.89 ±0.03 ^(e)^	1.92 ± 0.08 ^(g)^	0.82 ±0.05 ^(bcde)^	1.06 ± 0.10 ^(f)^	0.86 ±0.09 ^(de)^	0.61 ± 0.02 ^(a)^	1.10 ± 0.03 ^(f)^	0.69 ± 0.02 ^(ab)^	0.83 ± 0.02 ^(bcde)^	0.74 ± 0.05 ^(abcd)^	0.66
**CS ^2^**	**87**	**65**	**82**	**92**	**58**	**85**	**100**	**70**	**83**	**64**	**55**	**71**	**57**	**82**	**88**	
**Limiting AA**	SAA	SAA	SAA	SAA	SAA	SAA		Lys	Lys	Lys	Lys	Lys	Lys	Lys	Lys	

^1^ For EAA the following acronyms are used: His = histidine; Ile = isolucine; Leu = leucine; Lys = lysine; Met = methionine; Cys = cysteine; Phe = phenylalanine; Tyr = tyrosine; Thr = threonine; Val = valine; Trp = tryptofan. ^2^ CS = protein chemical score. * SAA, sulfur amino acids (Methionine + Cysteine); ** AAA, aromatic amino acids (Phenylalanine + Tyrosine).

**Table 4:** Sugar composition (mg/100 g) of gluten-free pasta samples. Different letters in columns indicate statistically significant differences (*p* < 0.05).

Sample	Galactose	Glucose	Fructose	Maltose	Total Reducing Sugars	Sucrose
*Gluten-Free Pasta—100% legume flour*						
P1	nd	12 ± 4 ^a^	25 ± 12 ^a^	92 ± 13 ^bc^	129	2753 ± 135 ^g^
P2	601 ± 22 ^d^	28 ± 10 ^a^	63 ± 17 ^a^	126 ± 02 ^c^	818	2880 ± 222 ^g^
P3	649 ± 91 ^d^	61 ± 3 ^a^	103 ± 45 ^ab^	315 ± 43 ^d^	1128	3332 ± 114 ^h^
P4	5 ± 2 ^a^	232 ± 76 ^b^	49 ± 5 ^a^	43 ± 0 ^ab^	329	3195 ± 187 ^h^
P5	234 ± 54 ^c^	27 ± 0.0 ^a^	25 ± 12 ^a^	120 ± 0 ^c^	406	2240 ± 111 ^f^
P6	93 ± 18 ^ab^	24 ± 5 ^a^	40 ± 8 ^a^	74 ± 3 ^abc^	231	3798 ± 156 ^i^
*Gluten-Free Pasta—Cereal, pseudocereal and legume flours (100% or mixed)*						
P7	3 ± 0.0 ^a^	52 ± 12 ^a^	36 ± 8 ^a^	89 ± 4 ^bc^	180	1430 ± 98 ^e^
P8	11 ± 3 ^a^	231 ± 32 ^b^	172 ± 45 ^bc^	25 ± 3 ^ab^	439	625 ± 14 ^ab^
P9	nd	318 ± 14 ^bc^	13 ± 2 ^a^	40 ± 32 ^ab^	371	637 ± 98 ^ab^
P10	7 ± 4 ^a^	252 ± 72 ^b^	183 ± 24 ^bcd^	15 ± 4 ^a^	467	762 ± 79 ^ab^
P11	189 ± 24 ^bc^	292 ± 67 ^bc^	293 ± 32 ^efg^	15 ± 7 ^a^	789	1071 ± 98 ^cd^
P12	nd	243 ± 54 ^b^	334 ± 78 ^fg^	553 ± 78 ^e^	1130	762 ± 45 ^ab^
P13	nd	311 ± 13 ^bc^	266 ± 23 ^def^	11 ± 5 ^a^	588	501 ± 25 ^a^
P14	181 ± 87 ^bc^	274 ± 45 ^bc^	212 ± 21 ^cde^	18 ± 9 ^a^	685	1196 ± 98 ^de^
P15	153 ± 21 ^bc^	400 ± 98 ^c^	365 ± 72 ^g^	6 ± 2 ^a^	924	888 ± 67 ^bc^
Mean	142	184	145	103	574	1738
Min-max	0–649	12–400	13–365	6–553	129–1130	501–3798

nd = not detectable.

**Table 5:** FUR, maltulose, HMF and AGPF in gluten-free pasta samples. Different letters in columns indicate statistically significant differences (*p* < 0.05).

Sample	FUR (mg/100 g Protein)	FUR (mg/100 g)	Maltulose (mg/100 g)	HMF (mg/kg)	AGPF (mg/kg)
*Gluten-free Pasta—100% legume flour*					
P1	21.0 ± 0.02 ^a^	4.6 ± 0.01 ^b^	5 ± 0 ^a^	0.099 ± 0.001	nd
P2	602.5 ± 2.79 ^i^	142.8 ± 0.66 ^g^	89 ± 10 ^c^	0.236 ± 0.003	nd
P3	787.6 ± 14.48 ^l^	169.3 ± 3.11 ^h^	125 ± 23 ^d^	0.147 ± 0.005	nd
P4	98.9 ± 1.26 ^d^	22.1 ± 0.28 ^d^	6 ± 4 ^a^	nd	nd
P5	244.6 ± 5.93 ^g^	64.3 ± 1.56 ^f^	32 ± 2 ^b^	nd	nd
P6	163.2 ± 0.61 ^f^	34.8 ± 0.16 ^e^	2 ± 0 ^a^	nd	nd
*Gluten-Free Pasta—Cereal, pseudocereal and legume flours (100% or mixed)*					
P7	18.9 ± 0.62 ^a^	1.6 ± 0.05 ^a^	2 ± 0 ^a^	0.106 ± 0.007	nd
P8	75.6 ± 0.05 ^b^	6.3 ± 0.01 ^b^	8 ± 1 ^a^	0.220 ± 0.001	nd
P9	65.2 ± 3.62 ^bc^	5.5 ± 0.30 ^b^	8 ± 5 ^a^	nd	nd
P10	82.7 ± 0.47 ^c^	5.6 ± 0.03 ^b^	6 ± 0 ^a^	nd	nd
P11	117.9 ± 2.00 ^l^	5.3 ± 0.09 ^b^	15 ± 2 ^ab^	nd	nd
P12	276.7± 9.84 ^h^	21.6 ± 0.776 ^d^	29 ± 4 ^b^	nd	nd
P13	78.3 ±0.78 ^bc^	5.2 ± 0.05 ^b^	9 ± 2 ^a^	nd	nd
P14	121.8 ± 1.55 ^e^	11.8 ± 0.15 ^c^	1 ± 0 ^a^	nd	nd
P15	129.8 ± 7.06 ^e^	13.9 ± 0.76 ^b^	4 ± 1 ^a^	nd	nd
Mean	192	34.3	23	-	-
Min-max	18.9–787.6	1.6–169.3	1–125	-	-

nd = not detectable.

## Data Availability

The data presented in this study are available on request from the corresponding author.

## Supplementary Materials

The following supporting information can be downloaded at: https://www.mdpi.com/article/10.3390/foods12061221/s1, Table S1: Instrumental conditions for chromatographic separation of amino acids.
