# Supplementary material for: Assessment of Nutritional Value and Maillard Reaction in Different Gluten-Free Pasta
Source: Foods. 2023 Mar 13;12(6):1221. doi: 10.3390/foods12061221 (PMC10048112; doi:10.3390/foods12061221)
Supplement: Supplementary file 1 [file foods-12-01221-s001.zip › foods-2249373-supplementary.pdf]

Table S1. Instrumental conditions for chromatographic separation of amino acids.

Mobile Phase (0.250 mL/min)				Time/Potential waveform		
**Time (min)**	**H_2O (%)**	**NaOH 250 mM (%)**	**NaOAc 1M (%)**	**Time (sec)**	**Potential (V)**	**Integration**
0.0	80	20	0	0.00	+ 0.13	
2.0	80	20	0	0.04	+ 0.13	
12.0	80	20	0	0.05	+ 0.28	
16.0	68	32	0	0.11	+ 0.28	start
24.0	36	24	40	0.12	+ 0.60	
40.0	36	24	40	0.41	+ 0.60	
40.1	20	80	0	0.42	+ 0.28	
42.1	20	80	0	0.56	+ 0.28	end
42.2	80	20	0	0.57	− 1.67	
62.0	80	20	0	0.58	− 1.67	
				0.59	+ 0.93	
				0.60	+ 0.13	
